# Assessing the Impact of Different Spirometric Equations on Asthma Severity and Control Among Children in Jordan: A Retrospective Study

**DOI:** 10.1155/pm/7562407

**Published:** 2025-09-19

**Authors:** Walid Al-Qerem, Anan Jarab, Judith Eberhardt, Enas Al-Zayadneh, Montaha Al-Iede, Lujain al-sa'di, Lama Sawaftah

**Affiliations:** ^1^Department of Pharmacy, Faculty of Pharmacy, Al-Zaytoonah University of Jordan, Amman, Jordan; ^2^Department of Clinical Pharmacy, Faculty of Pharmacy, Jordan University of Science and Technology, Irbid, Jordan; ^3^Department of Psychology, School of Social Sciences, Humanities and Law, Teesside University, Middlesbrough, UK; ^4^Department of Pediatrics, School of Medicine, University of Jordan, Amman, Jordan

**Keywords:** asthma, asthma control status, FEV_1_, Global Lung Initiative, lung function, spirometry

## Abstract

**Background:** Accurate assessment of lung function among asthmatic children is crucial for effective disease management. The Global Lung Initiative (GLI) has developed widely used spirometric reference equations. However, locally derived equations may better reflect regional population characteristics and more accurately predict asthma control status. The impact of using local versus GLI equations, particularly the newly developed race-neutral equations, remains under investigation. This study examined how the choice of spirometric equation affects asthma assessment.

**Method:** Spirometry was conducted on a sample of 438 asthmatic children (257 boys), and asthma control was assessed using the Global Initiative for Asthma Symptoms test (GINA-AST). Reference values, *z*-scores, and lower limits of normality (LLNs) were calculated for each child using both local and GLI reference equations. Concordance between equations was assessed using Cohen's kappa, and the sensitivity and specificity of each equation in detecting asthma control status were evaluated.

**Results:** Significant differences were found in spirometry values across equations. The local equation displayed the highest sensitivity for detecting uncontrolled asthma and showed the greatest agreement with GINA-AST. Mean FEV_1_*z*-scores varied across equations, though intraclass correlation coefficients (ICCs) were high.

**Conclusions:** This study highlights the substantial impact that the choice of spirometric equation has on asthma control assessment. Local equations may offer greater diagnostic sensitivity, potentially leading to more accurate disease classification and improved management outcomes.

## 1. Introduction

Asthma is a chronic respiratory disease that presents with a cluster of debilitating symptoms, including difficulty breathing, coughing, frequent respiratory infections, and wheezing [1]. It is widely recognized as the most common chronic disease among children worldwide [2, 3] profoundly impairing quality of life and placing a substantial burden on families and healthcare systems [4]. In addition to ranking among the Top 20 diseases worldwide in terms of disability-adjusted life years in children [5], the global prevalence of asthma is increasing rapidly. In Jordan, the estimated prevalence of asthma among children ranges from 8.8% to 9.5%, according to a cross-sectional study involving schoolchildren [6]. Additionally, the prevalence among Jordanian adolescents has been estimated at 12.3% [7].

Since asthma symptoms typically begin during school age, inadequate asthma management can have numerous adverse clinical and social consequences for affected children, including school absences, reduced quality of life, decreased physical activity, and recurrent hospitalizations. Moreover, previous research has shown that uncontrolled asthma may contribute to growth retardation [8]. Therefore, careful monitoring is essential when managing pediatric asthma. Yet no gold standard test currently exists to ensure optimal asthma control.

Spirometry is the most commonly used clinical test for evaluating lung function in both children and adults. It plays a crucial role in monitoring patients' response to therapy by detecting changes in lung function through measurements such as forced vital capacity (FVC) and forced expiratory volume in 1 s (FEV_1_), both of which are considered independent risk factors for exacerbation in asthmatic patients [9]. Various reference equations have been developed to interpret spirometric values, including the Global Lung Initiative 2012 (GLI2012) equations [10] and Al-Qerem equations for children from Middle Eastern populations [11]. These equations are based on age, sex, height, and ethnicity to provide accurate and representative predicted values. However, recent studies have raised concerns about the potential inaccuracy of estimating impaired lung function in certain populations when using ethnicity-specific reference equations [12, 13]. A prospective cohort study involving 6814 participants from diverse ethnic backgrounds found no evidence that race-specific reference equations improve the prediction of lung function [13]. Moreover, secondary analysis of several asthma observational studies concluded that using race-specific reference equations could mistakenly classify asthma as controlled when it is not—a misclassification that, if overlooked, could increase the risk of long-term complications [14]. The American Thoracic Society (ATS) has recommended replacing race-specific reference equations with race-neutral average equations, along with a more comprehensive evaluation of how these revised values are applied in clinical decision-making [15]. Given that treatment decisions are partly based on spirometry results, the Global Lung Initiative (GLI) developed internationally standardized race-neutral spirometric equations (GLI-2022) [16] in response to these concerns. Several studies have assessed the applicability of race-neutral equations and confirmed their validity across different populations [17]. However, findings also indicate that transitioning from race-specific to race-neutral equations can significantly alter the interpretation of spirometry results [18]. A US cohort study of 454 Black and White children evaluating race-norming in spirometry results showed significantly lower predicted FEV_1_ values in Black children when race-uncorrected equations were used, which emphasizes the variability between equations in estimating disease progression [19].

Accurate assessment of asthma control is critical for guiding treatment decisions, including whether to escalate or de-escalate therapy. However, these decisions depend heavily on the choice of spirometric reference equation, which can yield varying interpretations of lung function. Despite a growing interest in race-neutral approaches, the clinical implications of adopting such equations, particularly in comparison to locally derived reference standards, remain underexplored in many regions. Therefore, the present study aimed to evaluate the impact of using the GLI-2022 race-neutral equationsin comparison with the local Al-Qerem equations and the GLI-2012 race-specific equationson the assessment of asthma severity and control among Jordanian children with asthma.

## 2. Materials and Methods

This study is a part of a large project to assess various asthma aspects in Jordanian children [20]. The study involved 438 children undergoing asthma treatment at the Respiratory Therapy Unit of the University of Jordan Hospital (JUH). The inclusion criteria involved children under the age of 18 who were able to successfully complete a spirometry test and had a confirmed diagnosis of asthma by a pediatric pulmonologist. Medical records of children scheduled for appointments on the following day were reviewed in advance. During their visit, parents were approached in the waiting area and informed about the study. The interviewer provided a detailed explanation of the study's objectives, assured parents of the confidentiality of all collected data, and emphasized participants' right to withdraw at any time without consequences. Written informed consent was obtained from the parents of all participating children. This study was conducted in accordance with the ethical principles outlined in the Declaration of Helsinki.

### 2.1. Variables Analyzed in the Study

The primary variables collected for this study included sociodemographic, anthropometric, and clinical data. Sociodemographic information, specifically birthdate and sex (recorded as male or female), was obtained directly from each participant during enrollment, and birthdate together with test date was used to calculate each participant's age. As detailed in the Anthropometric Measures section, key physical characteristics were systematically measured and recorded, including height (centimeter), weight (kilogram), and the subsequently calculated body mass index (BMI). The main clinical outcome under investigation was asthma control status, which was evaluated using two complementary approaches. First, symptom-based control was assessed using the Global Initiative for Asthma Symptoms Test (GINA-AST), a standardized clinical tool routinely used for this purpose at the study hospital. This assessment is based on four items regarding symptoms over the past four weeks: (1) the presence of daytime symptoms more than twice a week, (2) the need for a SABA reliever more than twice a week, (3) any night waking due to asthma, and (4) any activity limitations due to asthma. Based on the number of items answered “yes,” patients were categorized as ‘well-controlled' (zero “yes” responses), ‘partially controlled' (one to two “yes” responses), or ‘uncontrolled' (three to four “yes” responses). For the dichotomous analysis in this study, the ‘partially controlled' and ‘well-controlled' groups were combined into a single ‘controlled' category, while the ‘uncontrolled' group remained classified as ‘uncontrolled'. Second, objective lung function was used to derive a control classification based on key spirometric parameters: FEV_1_, FVC, and the FEV_1_/FVC ratio. For the purposes of this analysis, asthma control was dichotomized into ‘controlled' or ‘uncontrolled' based on whether an individual's measured spirometric values were above or below the lower limit of normal (LLN) derived from each of the reference equations being compared.

### 2.2. Anthropometric Measures

Anthropometric measurements were conducted in a designated hospital room to ensure standardization. Participants were instructed to remove their shoes and any heavy clothing prior to assessment. Body weight was measured using a calibrated electronic scale and recorded to the nearest 0.1 kg. Height was measured using a stadiometer and rounded to the nearest centimeter. BMI was calculated using Quetelet's index (weight in kilograms divided by height in meters squared).

Spirometry was performed following the guidelines established by the ATS and the European Respiratory Society (ERS) [21]. The HDpft 3000 Pulmonary function testing system (nSpire health SPIROBANK II), equipped with disposable turbines, was used for all measurements. Children were seated upright with their feet flat on the floor to ensure proper posture for accurate assessment. A closed-circuit breathing maneuver was conducted using nasal clips.

To perform the test, children were instructed to seal their lips around the turbine, take a deep breath, and exhale forcefully for at least 6 s, or for a minimum of 3 s in children aged 10 years or younger. If a participant coughed or hesitated during the maneuver, particularly within the first second of exhalation, the test was repeated. The maneuver was also deemed unacceptable if any of the following occurred: incomplete exhalation, air leakage, obstruction in the turbine, or an extrapolated volume exceeding 5% of the FVC. A clear plateau in the volume-time curve was required for the maneuver to be considered valid.

To ensure consistency in measurement, the reproducibility criteria required the difference between the two highest FEV₁ and FVC values to be within 0.15 L. Participants were instructed to repeat the test up to eight times until three acceptable maneuvers were obtained. All spirometry assessments were conducted by the same investigator to maximize measurement accuracy and reliability.

### 2.3. Statistical Analysis

Data analysis was carried out using the Statistical Package for the Social Sciences (SPSS), Version 26. Descriptive statistics were computed: medians and interquartile ranges (25th–75th percentiles) were used for continuous variables, while frequencies and percentages were reported for categorical variables. Predicted values, *z*-scores, and LLNs for FEV_1_, FVC, and FEV_1_/FVC% were calculated using spirometry reference equations for children from a Middle Eastern population, as well as several GLI equations, namely, GLI 2012 GLI-O/Mixed (GLI-O) and GLI 2012 Caucasian (GLI-C), and the newly developed GLI race-neutral (GLI-N) equations.

Intraclass correlation coefficients (ICCs) were computed to assess agreement between *z*-scores produced by the different equations for each spirometric parameter. In addition, consistency in asthma control status classifications based on FEV_₁_, FVC, and FEV_₁_/FVC across the different equations was evaluated using Cohen's kappa and the McNemar test. Agreement between control status derived from FEV1 and FEV_1_/FVC using each equation and the GINA-AST was also assessed using Cohen's kappa. Furthermore, the diagnostic performance of spirometry-derived asthma control classifications was evaluated separately for males and females, using FEV_1_-LLN and FEV_1_/FVC-LLN thresholds from each equation in comparison to GINA-AST control status. Sensitivity, specificity, positive predictive value (PPV), and negative predictive value (NPV) were calculated to determine the accuracy of each equation in correctly identifying controlled and uncontrolled asthma.

The association between spirometry-based impairment in FEV_₁_, using the four studied equations, and GINA-AST control status was assessed with four equation-specific logistic regressions, in which GINA-AST control served as the dependent variable. All regressions were adjusted for age (years), sex (female/male), and BMI; age and BMI were *z*-standardized to improve numerical stability. Estimates are reported as adjusted odds ratios (aORs) with 95% confidence intervals. Because near-deterministic prediction is common when clinical control and spirometric impairment align closely, prespecified bias-reduced logistic regression (Firth penalized likelihood) was applied if separation was detected; otherwise, models were fit by maximum likelihood. To address multiplicity arising from the four primary FEV_₁_ coefficients (one per equation), Holm's method was used to control the family-wise error rate. Model diagnostics included checks for convergence, examination of influential observations, and inspection of variance inflation; analyses were conducted on complete cases.

A unified mixed-effects model was also explored to directly compare equations. However, the model showed evidence of quasi-complete separation, indicating a near-perfect predictive power of spirometry-based classifications on the GINA-AST outcome. This instability reinforces the exceptionally high concordance between the measures.

## 3. Results

Table 1 displays participants' sociodemographic characteristics. The sample included 438 children with asthma, the majority of whom were boys (58.7%). Among boys, the median age was 11.11 years (8.72–13.92), and the median BMI was 19.58 (16.45–23.72). For girls, the median age was 11.66 years (9.60–13.64), and the median BMI was 20.21 (16.88–24.61).

Table 2 displays *z*-scores, predicted percentages, LLNs, predicted values, and control status according to the different spirometric equations for females. The GLI-C equation produced the *z*-scores farthest from zero for both FEV_1_ and FVC, with medians of −0.766 (−1.796–0.205) and −0.455 (−1.32–0.276), respectively. In contrast, the Jordanian equation yielded the *z*-score farthest from zero for FEV_1_/FVC, with a median of −0.771 (−1.76–0.311). Furthermore, the highest LLNs for FEV_1_ and FEV_1_/FVC were observed with the Jordanian equation, with medians of 1.945 (1.53–2.226) and 79.915 (79.265–80.633), respectively, while the highest FVC LLN was produced by the GLI-C equation with a median of 2.178 (1.696–2.545).

The GLI-O equation resulted in the highest frequency of controlled FEV_1_ and FVC values, at 80.7% and 87.8%, respectively. Furthermore, the highest frequency of FEV_1_/FVC controlled values (76.8%) was observed for both the GLI-O and GLI-N equations.

For males, the GLI-C equation produced *z*-scores farthest from zero for both FEV_1_ and FVC, with medians of −0.902 (−1.755, −0.059) and −0.58 (−1.602–0.24), respectively. In contrast, the GLI-O equation yielded the *z*-score farthest from zero for FEV_1_/FVC, with a median of −0.566 (−1.628–0.462). The Jordan equation produced the highest LLNs for FEV_1_, FVC, and FEV_1_/FVC, with medians of 1.822 (1.414–2.505), 2.132 (1.635–2.862), and 75.005 (74.538–75.468), respectively.

GLI-O produced the highest frequency of controlled FEV_1_ status (81.3%), while both GLI-N and GLI-O yielded the highest frequency of controlled FVC status (87.8%). For FEV₁/FVC, the highest frequency of controlled status was observed with both the GLI-C and GLI-N equations (79.7%). In both females and males, high ICC values were reported for FEV_1_, FVC, and FEV_1_/FVC (Table 3).

The comparison of asthma control status across different equations is presented in Table 4. Among females, the greatest change in FEV_1_ control status occurred between the Jordan and GLI-O equations (8.8%), with a Cohen's kappa of 0.759. The smallest change was observed between the Jordan and GLI-C equations (2.2%), with a kappa of 0.945. For FVC, the highest change was between the GLI-C and the GLI-O equations (8.3%; *Kappa* = 0.700), while the lowest was between the GLI-O and GLI-N equations (1.7%) with a kappa of 0.927. Regarding FEV_₁_/FVC, the greatest change (5.5%) was observed in comparisons between both GLI-C versus GLI-O and GLI-O versus GLI-N, each with a kappa of 0.857. The smallest change was found between the GLI-C and GLI-N equations, with no observed difference (0%) and a perfect agreement (kappa = 1.00).

Among males, the greatest change in FEV₁ control status was observed between the Jordan and GLI-O equations (11.3%), with a kappa of 0.699. The smallest change occurred between the GLI-O and GLI-N equations, with a kappa of 0.950. For FVC, the highest change (9.8%) was seen in comparisons between both GLI-C versus GLI-N and GLI-C versus GLI-O equations, each with a kappa of 0.962. The smallest change (1.6%) was found between GLI-O and GLI-N, with a kappa of 0.937. Regarding FEV₁/FVC, the greatest change (5.9%) was observed between the Jordan and GLI-O equations, with a kappa of 0.828, while the smallest change (0%) occurred between GLI-C and GLI-N, indicating perfect agreement (kappa = 1.00).

Table 5 presents a comparison between GINA-AST control status and control status determined by different spirometric equations for both females and males. Among females, the greatest change in FEV_₁_ control status occurred with the GLI-O equation (32.6%; kappa = 0.243), while the smallest change was observed with the Jordan equation (31.5%; kappa = 0.301). Within the GINA-AST uncontrolled group, GLI-O showed the highest change (67.1%). For FEV₁/FVC, the largest change (37.6%) was observed with both GLI-N and GLI-C equations (*kappa* = 0.145), and the smallest change was with GLI-O (36.5%; kappa = 0.193). The highest change within the GINA-AST uncontrolled group was again seen with GLI-N and GLI-C (68.6%).

Among males, the greatest change in FEV₁ was observed with the GLI-O equation (40.9%; kappa = 0.250) and the smallest with the Jordan equation (32.7%; kappa = 0.301). In the GINA-AST uncontrolled group, GLI-O also showed the highest change (69.4%). The largest change in FEV_₁_/FVC was with GLI-O (45.4%; kappa = 0.147), while the smallest was with both GLI-N and GLI-C (44.4%; kappa = 0.181). The highest change in the GINA-AST uncontrolled group was observed with the Jordan equation (72.6%).

Sensitivity, specificity, PPV, and NPV analyses indicated that, across both sexes, the FEV_₁_ Jordan control classification exhibited the highest sensitivity (47.6% in males and 45.7% in females), suggesting it was the most effective at detecting cases of uncontrolled asthma. Conversely, the FEV_1_ GLI-O and GLI-N equations demonstrated the highest specificity (97.3% in males and 89.2% in females), indicating their superior ability to correctly identify controlled cases and minimize false positives. PPV was consistently highest for FEV_1_ GLI-O (93.8% in males and 65.7% in females), suggesting that when this equation classified a case as uncontrolled, it was the most accurate in doing so. The FEV_1_ Jordan equation showed the highest NPV (57.2% in males and 70.8% in females), making it the most reliable for confirming asthma control.

While FEV_1_-based equations generally outperformed FEV_1_/FVC-based equations, the latter demonstrated moderate diagnostic accuracy, particularly among females. In this group, the FEV_1_/FVC Jordan equation showed higher sensitivity (35.7%), but lower specificity (81.9%) compared to the FEV_1_ models. These findings highlight notable sex-based differences in asthma control classification, with males exhibiting higher specificity across all equations, whereas females demonstrated slightly better sensitivity and NPV. Overall, the results suggest that the FEV_₁_ Jordan equation is optimal for detecting uncontrolled asthma, while FEV_1_ GLI-O and GLI-N are preferable for minimizing misclassification of controlled cases. These distinctions may support more informed clinical decisions on asthma management based on sex-specific diagnostic performance.

Across four equation-specific multivariable logistic models, FEV₁ classified as “uncontrolled” was strongly and consistently associated with clinical uncontrolled asthma by GINA-AST, with aORs ranging from 5.62 (95% CI 3.30–9.84; GLI-C) to 7.57 (4.36–13.63; Jordan). The corresponding effects for GLI-N and GLI-O were 6.65 (3.57–13.06) and 6.18 (3.29–12.34), respectively; all remained significant after Holm adjustment across the four primary tests (all adjusted *p* < 0.001). Male sex showed a similar independent association in every model (aOR ≈ 1.8–1.9, *p* ≈ 0.01), whereas age and BMI (*z*-scores) were not materially related to clinical control (all *p* > 0.30) (Table 6).

## 4. Discussion

This study compared the impact of different spirometric equations, including the local Al-Qerem equations, the newly developed GLI-2022 race-neutral reference equations, as well as two distinct GLI-2012 race-specific equations (GLI-2012 Mixed [GLI-O] and GLI-2012 Caucasian [GLI-C]) on the assessment of asthma severity classification and control status among Jordanian children with asthma. The results indicated that these equations produced varying predicted lung function values, with notable discrepancies between males and females. Our findings highlight the importance of using the most appropriate reference equation for the Jordanian population to ensure consistent interpretation of lung function tests.

The local Al-Qerem reference equations for Jordanian children were analyzed alongside several GLI equations selected for their applicability to the population studied. For instance, the GLI/O-mixed equations, which are recommended for populations underrepresented in the GLI database, were included. The GLI-C equations, which reflect values for individuals of Middle Eastern origin, were also used. Additionally, the study incorporated the recently developed GLI-2022 race-neutral equations, which were designed to address concerns regarding the potential inaccuracies of race-specific GLI-2012 equations in estimating lung function.

Comparisons between the control status across the different spirometric equations revealed notable inconsistencies. The greatest discordance in FEV_1_ control status was observed between the Al-Qerem and GLI-O equations, particularly among males. This finding suggests that reliance on certain GLI equations may lead to overestimation or underestimation of asthma severity, potentially influencing clinical decision-making. Notably, the GLI-2022 race-neutral equations produced results broadly comparable to those of the GLI-2012 Caucasian and GLI-O equations, albeit with moderate differences in z-score distributions and lung function classification. These discrepancies emphasize the need for cautious interpretation when applying global reference equations to diverse populations.

Similar variations have been reported in studies comparing race-neutral and race-specific equations across different ethnic groups. For example, a multicenter cross-sectional US study comparing GLI-2022 with GLI-2012 equations revealed that transitioning to the newer equations significantly affected lung function interpretation, with the highest percentage of changes being observed among Black patients [18]. Likewise, an observational cross-sectional study of 886 healthy Chinese children showed significant differences across almost every spirometric measure, particularly FEV_1_*z*-scores, which deviated in opposite directions: GLI-2022 yielded negative mean *z*-scores for both sexes, while GLI-2012 produced positive values. This pattern suggests potential overestimation by the race-neutral equations [17]. Together, these findings highlight the ongoing debate surrounding the standardization of lung function assessment and the applicability of global reference equations to ethnically diverse populations.

Based on the comparison of asthma control status determined by the studied spirometry equations and GINA-AST, the findings demonstrated that the Al-Qerem equation exhibited the highest sensitivity among all equations, with the lowest proportion of cases classified as controlled and the highest Cohen's kappa value. This indicates fair agreement between the two methods and highlights the Al-Qerem equation's superior ability to correctly identify uncontrolled asthma. These results are further supported by its highest NPV, which represents the proportion of individuals with negative test results who truly do not have the condition [22].

Although the Al-Qerem equation did not show the highest percentage change in FEV_₁_/FVC control status compared to other equations, this limitation is of less clinical significance, as FEV_₁_ has been identified in numerous studies as a stronger predictor of disease control and exacerbation risk [23]. In our study, FEV_1_/FVC displayed lower specificity than the FEV_1_ models. The enhanced sensitivity of the Al-Qerem equation may be attributed to its derivation from a large, representative sample of the Jordanian population, providing a better population-specific fit. Moreover, it addresses key limitations of the GLI-2012 equations, particularly those related to population diversity and evolving anthropometric profiles.

A prospective cohort study of 2219 patients with Interstitial Lung Disease (ILD) [24] evaluating the application of GLI equations found that 1291 patients were classified as having normal pulmonary function despite a confirmed ILD diagnosis. This discrepancy may be attributed to the higher sensitivity of locally derived equations when applied to specific clinical populations, such as ILD patients. Similarly, a study involving 3804 Italian children concluded that the GLI reference equations did not provide an optimal fit for that population, resulting in a lower probability of detecting lung function decline [25]. Additionally, a cross-sectional study assessing the applicability of the GLI-2012 equations in a Tunisian population reported significantly higher FEV_1_ and FEV_1_/FVC values using the GLI equations compared to those produced by local Tunisian equations. Consequently, the authors did not support the use of GLI equations for clinical decision-making in that setting [26]. However, it is important to note that while local equations may offer greater accuracy for smaller, well-defined populations, their sensitivity may diminish when applied to more diverse or heterogeneous groups [18, 27].

On the other hand, the highest specificity was observed with the GLI-O and GLI-N equations, indicating their superiority in minimizing false positives and accurately classifying controlled cases. A potential explanation is that the GLI equations yield higher predicted lung function values than the local equations; as a result, only patients with substantial impairment fall below the predicted normal range, leading to higher specificity. Our findings support this interpretation: FEV_1_ predicted percentage values were notably higher using GLI-O and GLI-N equations compared to the local equations. For example, among females, FEV_₁_ predicted% values were 95.96% for GLI-O, 92.07% for GLI-N, and 89.45% for the Jordan equation.

Similar results have been reported in other regions. A retrospective analysis of data from 406 Canadian participants compared a local Canadian reference equation with the GLI-2012 equations. A higher prevalence of restrictive lung disease was observed using the local equation, suggesting that the GLI identifies fewer individuals as impaired, a finding that may reflect their higher specificity [28]. In contrast, a recent study from Norway assessing the use of GLI reference equations versus the widely used European Coal and Steel Community (ECSC) equation showed that mean predicted FEV_1_ percentage was five percentage points lower when using the GLI equations. This difference was found to affect disease grading [29], resulting in fewer patients falling below the LLN when transitioning from ECSC to GLI.

Interestingly, notable sex-based differences in asthma control classification using the Al-Qerem equation were observed despite incorporating sex-specific adjustments. Specifically, females demonstrated higher sensitivity, while males exhibited higher specificity across all equations. Complementing this finding, our multivariable regression analysis showed a modest but consistent increase in the odds of clinically uncontrolled status among boys, an effect that persisted after adjusting for age and BMI. These differences may be attributed to anatomical and environmental factors that vary between the sexes [30], which have not been thoroughly investigated in pediatric populations. Supporting this, a cohort study involving children with both severe asthma and identified a male-specific association between exacerbation risk and lower FEV_1_ values [31]. Taken together with our findings, this suggests that asthma control may be improved by implementing sex-specific early interventions.

The differences observed between equations used to assess asthma control are likely to influence clinical decision-making and may result in significant variation in asthma management. These variations can occur through overestimating or underestimating lung function, potentially leading to withholding important interventions, depending on several factors, most importantly, the population being studied. Moreover, the existence of multiple applicable equations for the same population may lead to inconsistent clinical judgments, particularly if hospitals and clinics adopt different reference standards. This highlights the urgent need to develop equitable tools that accurately represent diverse populations and support consistent, evidence-based care.

Taken together, while the local and GLI equations differ in their diagnostic thresholds for sensitivity and specificity, the regression analysis confirms the fundamental clinical signal remains robust. Children flagged as “uncontrolled” by an FEV₁ measurement were far more likely to be clinically uncontrolled by GINA symptoms, and this powerful association persisted regardless of which reference equation was used. This finding is consistent with recent literature suggesting that while newer equations like GLI-2022 may alter interpretive thresholds, the underlying link between poor lung function and poor clinical control remains unchanged [32]. However, this underlying robustness does not diminish the clinical importance of selecting the appropriate equation, as this choice directly determines which children are identified as having impaired lung function and are therefore considered for changes in therapy.

### 4.1. Strengths, Limitations, and Future Directions

Despite the valuable comparisons provided by our study, several limitations should be acknowledged. First, the reliance on data from a single population in Jordan may limit its broader applicability. A multicenter approach would likely yield more representative results across diverse populations and clinical settings. Second, the observational cross-sectional design limits the ability to establish causality. Future research using a longitudinal design could provide deeper insights into disease progression and equation performance over time. Finally, unmeasured confounding variables, such as environmental exposures, socioeconomic status, or body composition, may have influenced the results.

On the other hand, our study has several strengths. To our knowledge, it is the first study in Jordan to compare the local Al-Qerem equation with multiple GLI equations in the assessment of asthma severity and control. The sample size was adequate, enhancing the statistical power and reliability of our findings. Additionally, data collection was carried out using a modern spirometer known for its accuracy, which stores spirometry results and anthropometric data digitally, thereby reducing human error and eliminating the need for manual data transfer. Importantly, all spirometry tests were conducted by the same trained technician using the same device, ensuring procedural consistency.

## 5. Conclusion

This study evaluated the impact of applying different spirometry equations on the assessment of asthma control among patients. The findings revealed notable discrepancies between the local and GLI equations in predicted lung function values, which, in turn, affected asthma control classification and the overall clinical decision-making process. These results highlight the potential for overestimation or underestimation of asthma severity when using GLI equations and support the superiority of locally derived equations, such as the Al-Qerem equation, for the designated population.

## Figures and Tables

**Table 1:** Sociodemographic characteristics of the study participants.

	**Median (25th–75th percentiles)**	
	**Boys 257 (57.63%)**	**Girls 181 (42.37%)**
Age	11.11 (8.72–13.92)	11.66 (9.60–13.64)
Height	144 (133–159)	147(138–157)
FVC	2.36 (1.89–3.13)	2.37 (1.86–2.97)
FEV_1_	1.95 (1.57–2.56)	2.06 (1.66–2.68)
FEV_1_/FVC	84.28 (77.78–90.44)	86.42 (79.07–92.82)
BMI	19.58 (16.45–23.72)	20.21 (16.88–24.61)

**Table 2:** *Z*-scores, predicted percentages, lower limits of normal, predicted values, and control status based on different spirometric equations for females.

		**GLI-C**	**GLI-N**	**GLI-O**	**Jordan**	**ICC**
	**Median (25–75 percentiles)**					
FEV_1_*z*-score		−0.766 (−1.796–0.205)	−0.366 (−1.414–0.468)	−0.201 (−1.301–0.844)	−0.681 (−1.825–0.391)	0.975
FVC *z*-score		−0.455 (−1.32–0.276)	−0.114 (−0.925–0.56)	0.253 (−0.74–1.078)	−0.278 (−1.114–0.491)	0.980
FEV_1_/FVC *z*-score		−0.563 (−1.507–0.746)	−0.645 (−1.538–0.67)	−0.768 (−1.757–0.583)	−0.771 (−1.76–0.311)	0.997
FEV_1_ predicted %		90.953 (78.713–102.397)	95.207 (81.389−105.956)	97.626 (84.488−109.909)	92.105 (80.243−104.841)	
FVC predicted %		94.647 (84.191−103.272)	98.455 (87.807−107.48)	102.869 (91.505−112.243)	96.494 (86.874−106.613)	
FEV_1_/FVC predicted %		96.246 (89.049−104.095)	95.835 (88.804−103.792)	95.231 (88.11−102.998)	0.949 (0.869−1.019)	
FEV lower limit of normal		1.929 (1.492−2.242)	1.777 (1.441−2.082)	1.792 (1.386−2.083)	1.945 (1.53−2.226)	
FVC lower limit of normal		2.178 (1.696−2.545)	1.966 (1.612−2.371)	2.026 (1.579−2.368)	2.139 (1.668−2.48)	
FEV_1_/FVC lower limit of normal		0.784 (0.781−0.789)	0.788 (0.782−0.793)	0.804 (0.8−0.808)	79.915 (79.265−80.633)	
FEV_1_ predicted		2.395 (1.854−2.784)	2.28 (1.835−2.669)	2.231 (1.727−2.594)	2.372 (1.866−2.715)	
FVC predicted		2.695 (2.099−3.151)	2.506 (2.057−3.033)	2.48 (1.931−2.899)	2.628 (2.049−3.046)	
FEV_1_/FVC predicted		0.893 (0.889−0.899)	0.897 (0.89−0.903)	0.903 (0.899−0.909)	90.897 (90.158−91.713)	
FEV_1_ control	Uncontrolled	49 (27.1%)	37 (20.4%)	35 (19.3%)	51 (28.2%)	
	Controlled	132 (72.9%)	144 (79.6%)	146 (80.7%)	130 (71.8%)	
FVC control	Uncontrolled	37 (20.4%)	25 (13.8%)	22 (12.2%)	30 (16.6%)	
	Controlled	144 (79.6%)	156 (86.2%)	159 (87.8%)	151 (83.4%)	
FEV1/FVC control	Uncontrolled	42 (23.2%)	42 (23.2%)	52 (28.7%)	48 (26.5%)	
	Controlled	139 (76.8%)	139 (76.8%)	129 (71.3%)	133 (73.5%)	

**Table 3:** *Z*-scores, predicted percentages, lower limits of normal, predicted values, and control status based on different spirometric equations for males.

		**GLI-C**	**GLI-N**	**GLI-O**	**Jordan**	**ICC**
	**Median (25–75 percentiles)**					
FEV_1_*z*-score		−0.902 (−1.755–−0.059)	−0.622 (−1.409–0.313)	−0.341 (−1.257–0.565)	−0.873 (−1.929–0)	0.985
FVC *z*-score		−0.58 (−1.602–0.24)	−0.254 (−1.134–0.622)	0.074 (−1.053–1.05)	−0.481 (−1.585–0.241)	0.979
FEV_1_/FVC *z*-score		−0.378 (−1.389–0.583)	−0.452 (−1.434–0.534)	−0.566 (−1.628–0.462)	−0.515 (−1.36–0.275)	0.996
FEV_1_ predicted %		89.396 (79.36–99.261)	92.073 (81.785–103.927)	95.955 (85.183–106.543)	89.451 (79.013–100.003)	
FVC predicted %		92.827 (81.111–102.782)	96.567 (85.1–108.115)	100.81 (88.086–111.622)	93.789 (81.656–103.366)	
FEV_1_/FVC predicted %		97.298 (89.112–104.009)	96.794 (89.015–103.498)	96.272 (88.172–102.912)	95.838 (87.891–102.073)	
FEV LLN		1.805 (1.441–2.422)	1.689 (1.378–2.274)	1.677 (1.339–2.25)	1.822 (1.414–2.505)	
FVC LLN		2.11 (1.659–2.842)	1.963 (1.583–2.632)	1.966 (1.546–2.646)	2.132 (1.635–2.862)	
FEV_1_/FVC LLN		0.756 (0.75–0.765)	0.759 (0.753–0.767)	0.775 (0.769–0.783)	75.005 (74.538–75.468)	
FEV_1_ predicted		2.225 (1.786–3.018)	2.145 (1.754–2.887)	2.073 (1.664–2.812)	2.249 (1.763–3.056)	
FVC predicted		2.604 (2.057–3.498)	2.496 (2.013–3.345)	2.398 (1.894–3.221)	2.63 (2.017–3.53)	
FEV_1_/FVC predicted		0.867 (0.861–0.877)	0.871 (0.863–0.879)	0.876 (0.87–0.886)	88.379 (87.56–89.063)	
FEV_1_ control	Uncontrolled	74 (28.8%)	52 (20.2%)	48 (18.7%)	77 (30%)	
	Controlled	183 (71.2%)	205 (79.8%)	209 (81.3%)	180 (70%)	
FVC control	Uncontrolled	62 (24.2%)	37 (14.5%)	37 (14.5%)	56 (21.9%)	
	Controlled	194 (75.8%)	219 (85.5%)	219 (85.5%)	200 (78.1%)	
FEV_1_/FVC control	Uncontrolled	52 (20.3%)	52 (20.3%)	63 (24.6%)	48 (18.8%)	
	Controlled	204 (79.7%)	204 (79.7%)	193 (75.4%)	208 (81.3%)	

**Table 4:** Comparison of asthma control status across different equations.

		**GLI-C vs. GLI-N**	**GLI-C vs. GLI-O**	**Jordan vs. GLI-C**	**Jordan vs. GLI-N**	**Jordan vs. GLI-O**	**GLI-O vs. GLI-N**
*Females*							
FEV_1_	Change frequency (%)	12 (6.6%)	14 (7.7%)	4 (2.2%)	14 (7.7%)	16 (8.8%)	6 (3.3%)
	Kappa (MCNEMAR)	0.818 (< 0.001)	0.785 (< 0.001)	0.945 (0.63)	0.792 (< 0.001)	0.759 (< 0.001)	0.896 (0.69)
FVC	Change frequency (%)	12 (6.6%)	15 (8.3%)	7 (3.9%)	9 (5%)	8 (4.4%)	3 (1.7%)
	Kappa (MCNEMAR)	0.768 (< 0.001)	0.7 (< 0.001)	0.872 (0.016)	0.807 (0.18)	0.821 (0.008)	0.927 (0.25)
FEV_1_/FVC	Change frequency (%)	0 (0%)	10 (5.5%)	6 (3.3%)	6 (3.3%)	4 (2.2%)	10 (5.5%)
	Kappa (MCNEMAR)	1 (1.00)	0.857 (0.002)	0.911 (0.031)	0.911 (0.031)	0.945 (0.125)	0.857 (0.002)

*Males*							
FEV_1_	Change frequency (%)	22 (8.6%)	26 (10.1%)	15 (5.8%)	27 (10.5%)	29 (11.3%)	4 (1.6%)
	Kappa (MCNEMAR)	0.771 (< 0.001)	0.724 (< 0.001)	0.859 (0.607)	0.724 (< 0.001)	0.699 (< 0.001)	0.950 (0.125)
FVC	Change frequency (%)	25 (9.8%)	25 (9.8%)	10 (3.9%)	21 (8.2%)	19 (7.4%)	4 (1.6%)
	Kappa (MCNEMAR)	0.692 (< 0.001)	0.692 (< 0.001)	0.89 (0.11)	0.727 (< 0.001)	0.753 (< 0.001)	0.937 (1.00)
FEV_1_/FVC	Change frequency (%)	0 (0%)	11 (4.3%)	4 (1.6%)	4 (1.6%)	15 (5.9%)	11 (4.3%)
	Kappa (MCNEMAR)	1 (1.00)	0.877 (0.001)	0.95 (0.125)	0.95 (0.125)	0.828 (< 0.001)	0.877 (0.001)

**Table 5:** Comparison of GINA-AST control status with the control status derived from different spirometric equations.

		**GINA-AST control status**		**Kappa**
		**Uncontrolled**	**Controlled**	
		**Change count (%)**		
*Females*				
FEV_1_	Jordan vs. GINA-AST	38 (54.3%)	19 (17.1%)	0.301
	GLI-N vs. GINA-AST	46 (65.7%)	13 (11.7%)	0.247
	GLI-C vs. GINA-AST	39 (55.7%)	18 (16.2%)	0.297
	GLI-O vs. GINA-AST	47 (67.1%)	12 (10.8%) 13%	0.243
FEV_1_/FVC	Jordan vs. GINA-AST	45 (64.3%)	23 (20.7%)	0.159
	GLI-N vs. GINA-AST	48 (68.6%)	20 (18%)	0.145
	GLI-C vs. GINA-AST	48 (68.6%)	20 (18%)	0.145
	GLI-O vs. GINA-AST	42 (60%)	24 (21.6%)	0.193

*Males*				
FEV_1_	Jordan vs. GINA-AST	77 (52.4%)	7 (6.4%)	0.382
	GLI-N vs. GINA-AST	98 (66.7%)	3 (2.7%)	0.276
	GLI-C vs. GINA-AST	81 (55.1%)	8 (7.3%)	0.347
	GLI-O vs. GINA-AST	102 (69.4%)	3 (2.7%)	0.250
FEV_1_/FVC	Jordan vs. GINA-AST	106 (72.6%)	8 (7.3%)	0.181
	GLI-N vs. GINA-AST	104 (71.2%)	10 (9.1%)	0.178
	GLI-C vs. GINA-AST	104 (71.2%)	10 (9.1%) 44.4	0.178
	GLI-O vs. GINA-AST	100 (68.5%)	17 (15.5%) 45.5	0.147

**Table 6:** Multivariable associations between FEV1 status and GINA-AST control status, by equation⁣^∗^.

**Equation**	**Predictor**	**Adjusted OR**	**95% CI**	**p**	**p** **(Holm, FEV**_**1**_ ** only)**
Jordan	FEV1 uncontrolled (vs. controlled)	7.57	4.36–13.63	< 0.001	< 0.001
	Age (*z*-score)	1.02	0.76–1.37	0.905	
	Male (vs. female)	1.87	1.13–3.13	0.016	
	BMI (*z*-score)	0.99	0.75–1.32	0.956	

GLI-N	FEV1 uncontrolled (vs. controlled)	6.65	3.57–13.06	< 0.001	< 0.001
	Age (*z*-score)	1.15	0.87–1.54	0.325	
	Male (vs. female)	1.92	1.18–3.17	0.009	
	BMI (*z*-score)	0.97	0.73–1.27	0.810	

GLI-C	FEV1 uncontrolled (vs. controlled)	5.62	3.30–9.84	< 0.001	< 0.001
	Age (*z*-score)	1.11	0.84–1.48	0.462	
	Male (vs. female)	1.83	1.12–3.01	0.017	
	BMI (*z*-score)	0.96	0.73–1.26	0.764	

GLI-O	FEV1 uncontrolled (vs. controlled)	6.18	3.29–12.34	< 0.001	< 0.001
	Age (*z*-score)	1.11	0.84–1.48	0.462	
	Male (vs. female)	1.84	1.14–3.01	0.014	
	BMI (*z*-score)	0.96	0.73–1.27	0.788	

⁣^∗^Separate logistic regressions per equation with GINA clinical control (uncontrolled vs. controlled) as the outcome, adjusted for age, sex, and BMI. Primary effect per model is the FEV_1_ uncontrolled vs. controlled coefficient. Holm adjustment applied across the four primary tests.

## Data Availability

The dataset supporting the conclusions of this article is available in the Zenodo repository: 10.5281/zenodo.15049475.
